# The Role of Hyperbaric Oxygen Therapy in the Treatment of Surgical Site Infections: A Narrative Review

**DOI:** 10.3390/medicina59040762

**Published:** 2023-04-14

**Authors:** Dingzi Zhou, Daigang Fu, Ling Yan, Linshen Xie

**Affiliations:** West China School of Public Health, West China Fourth Hospital, Sichuan University, Chengdu 610041, China

**Keywords:** hyperbaric oxygen therapy, surgical site infections, treatment, reactive oxygen species

## Abstract

Surgical site infections (SSIs) are among the most prevalent postoperative complications, with significant morbidity and mortality worldwide. In the past half century, hyperbaric oxygen therapy (HBOT), the administration of 100% oxygen intermittently under a certain pressure, has been used as either a primary or alternative therapy for the management or treatment of chronic wounds and infections. This narrative review aims to gather information and evidence supporting the role of HBOT in the treatment of SSIs. We followed the Scale for the Quality Assessment of Narrative Review Articles (SANRA) guidelines and scrutinized the most relevant studies identified in Medline (via PubMed), Scopus, and Web of Science. Our review indicated that HBOT can result in rapid healing and epithelialization of various wounds and has potential beneficial effects in the treatment of SSIs or other similar infections following cardiac, neuromuscular scoliosis, coronary artery bypass, and urogenital surgeries. Moreover, it was a safe therapeutic procedure in most cases. The mechanisms related to the antimicrobial activity of HBOT include direct bactericidal effects through the formation of reactive oxygen species (ROS), the immunomodulatory effect of HBOT that increase the antimicrobial effects of the immune system, and the synergistic effects of HBOT with antibiotics. We emphasized the essential need for further studies, especially randomized clinical trials and longitudinal studies, to better standardize HBOT procedures as well as to determine its full benefits and possible side effects.

## 1. Introduction

Surgical site infections (SSIs) are among the most prevalent postoperative complications, responsible for significant morbidity and mortality, delayed wound healing, increased length of hospital stay, unnecessary pain, and a high cost to the patient and the institution [1,2,3]. SSIs are the most common hospital-associated infections (HAI) in low- or middle-income countries, affecting up to a third of patients who had surgery, as well as being a frequent HAI in high-income countries in the European Union and the USA [4,5]. The risk of developing an SSI is multifactorial, and several intrinsic (patient) and extrinsic (e.g., procedure, facility, preoperative, and operative) risk factors have been determined for the development or incidence of SSIs [6]. The intrinsic factors include increasing age, diabetes mellitus, radiation, immunosuppression, history of skin or soft tissue infection, obesity, use of alcohol, smoking, dyspnea, low serum albumin concentration, and total bilirubin > 1.0 mg/dL [6,7,8]. The extrinsic factors comprise emergency and more complex surgery, inadequate ventilation, inappropriate sterilization of equipment, increased operating room traffic, the presence of a pre-existing infection, inadequate skin preparation or hair removal, inappropriate or no antibiotic prophylaxis, blood transfusion, the length of the operation, the duration of the surgical scrub, the maintenance of asepsis, poor-quality surgical hand scrubbing and gloving, hypothermia, and poor glycemic control [6,7,9].

Despite significant progress in the control of infectious diseases in hospitals, SSIs are still among the most prevalent postoperative problems. The prevention and management of SSIs are complex, and an integration of pre-, intra-, and post-operative measures is required to reduce the burden and complications of SSIs [5,10,11]. Antibiotic prophylaxis and treatments are wildly used in the prevention and management of SSIs following surgeries [11,12], and have a critical role in the achievement of a better clinical outcome for wound healing and SSIs [11,13]. Unfortunately, antibiotic resistance in microorganisms has continuously increased over the past decades, and antibiotic efficacy has decreased for many pathogens [14,15]. Recent data showed that more than 50% of the pathogens isolated from SSIs were bacterial pathogens that were multidrug-resistant organisms (MDROs) [16,17]. Increasing trends in antibiotic resistance potentially threaten the safety and efficacy of surgical procedures; therefore, the development of alternative treatments is essentially needed to reduce the amount of antibiotic usage and better manage SSIs [18]. Various therapy procedures using oxygen, including local oxygen therapy, supplemental inspired oxygen therapy, and hyperbaric oxygen therapy, have shown beneficial impacts on wound healing and reduced the burden of SSIs [19,20,21].

Hyperbaric oxygen therapy (HBOT) is a promising treatment modality, as either a primary or alternative therapy, for the management of some complex medical conditions [22,23,24] including non-healing wounds [25,26] as well as various hypoxic or ischemic events [27,28]. Moreover, HBOT has a potential therapeutic effect which could be used for the treatment of acute infections caused by MDROs [29,30,31]. This narrative review aims to gather current and comprehensive information on the clinical efficacy, mechanisms of action, and complications of HBOT for the treatment of SSIs.

## 2. HBOT Procedure

**Figure 1:**
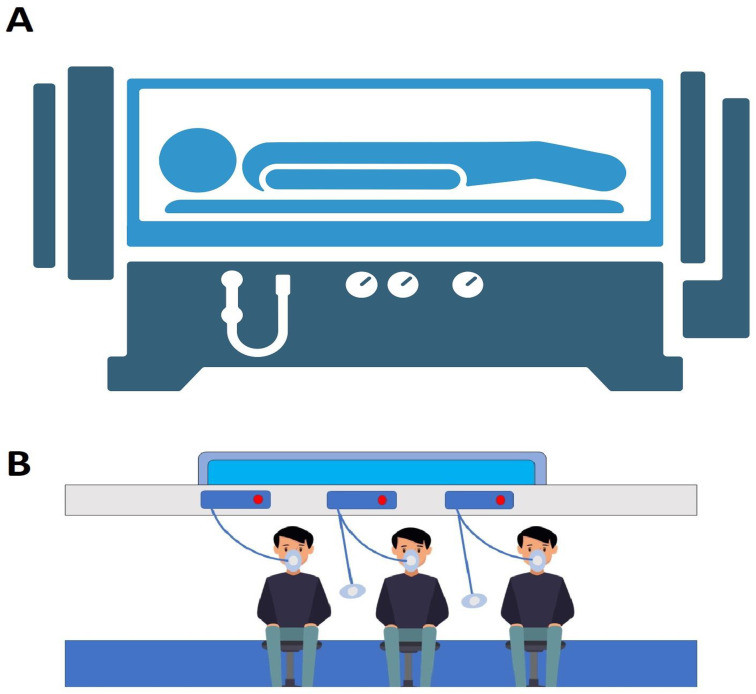
Hyperbaric oxygen therapy (HBOT) procedure. (**A**) illustration of a monoplace chamber (**B**) illustration of a multiplace chamber.

HBOT is a treatment based on the administration of 100% oxygen (pure O_2_) intermittently at a higher-than-normal atmospheric pressure inside a specially designed monoplace or multiplace chamber [32,33,34]. A monoplace chamber holds only a single patient who breathes pressurized pure O_2_ directly, while in a multiplace chamber there are two or more patients who breathe pure O_2_ indirectly through head hoods, masks, or endotracheal tubes (Figure 1) [18,32]. In multiplace chambers, patients can access a caregiver in the chamber and receive hands-on care during their treatment periods [34]. HBOT differs from topical oxygen therapy (TOT). TOT is the administration of pure O_2_ under pressure to a particular injured tissue [35], while a patient must receive the oxygen by inhalation within a pressurized chamber at a pressure of 1.4 atmosphere absolute or higher for this to be considered HBOT [36]. Generally, the time period for the majority of the elective HBOTs is about 90 min and runs between 2 and 3 absolute atmosphere pressure depending upon the therapeutic effects desired; however, for urgent treatments, it may run longer and at greater pressures [34]. HBOT is considered an opportunity for clinicians to better treat and manage several clinical problems, including carbon monoxide poisoning, ischemia, inflammation, acute wounds, and infections [18,34].

## 3. Clinical Application of HBOT

Currently, HBOT is considered either alone or as an adjunct treatment for several cute or chronic diseases [33,37,38,39]. It is suggested to treat ocular disorders, including cystoid macular edema, scleral thinning, and necrosis faced after pterygium surgery; nonhealing corneal edema; anterior segment ischemia; and some blinding diseases [37,40]. Several experimental and clinical studies also showed a beneficial effect from HBOT in brain/cerebral injuries [41,42,43]. Animal studies have shown the inhibitory effect of HBOT on inflammation and apoptosis after cerebral ischemia [41,44]. Furthermore, these studies have shown that HBOT is associated with reduced blood–brain barrier breakdown, reduced cerebral edema, improved cerebral oxygenation, decreased intracranial pressure, reduced oxidative burden, reduced metabolic derangement, and increased neural regeneration [41,42,45]. Clinical trials on humans have not shown any significant benefit, although it is indicated that HBOT can improve some neuropsychological and inflammatory outcomes, especially in stroke patients, within the first few hours [42,46]. Moreover, there is evidence suggesting that HBOT is not only safe for cancer patients, but might also have tumor-inhibitory effects in certain cancer subtypes and can also be used in the treatment of complications after radiotherapy [33,47]. Most importantly, HBOT is used in the treatment of acute or chronic wounds, diabetic foot ulcers, and infectious diseases [18,48,49,50,51,52].

## 4. Methods

This narrative review was performed according to the Scale for the Quality Assessment of Narrative Review Articles (SANRA) guideline [53]. To find the most relevant studies, we searched four international scientific databases—PubMed, Scopus, Web of Science and SciELO—for peer-reviewed articles published before 30 December 2022. The literature search was conducted using the following keywords and related MESH terms, employing the Boolean operators “AND” and/or “OR”: hyperbaric oxygen therapy, surgical site infections, wound infection, sternal wound infection. We did not apply any time and language restrictions, and the abstracts and key parts of papers published in languages other than English were translated into English using ‘Google Translate’. After the duplicates were removed, the titles and abstracts of the retrieved studies were screened to select potentially relevant articles. The full texts of the remaining studies were reviewed in depth to determine whether they met the established inclusion criteria. All types of studies evaluating the effects of HBOT in the treatment of SSIs, including case studies, were included. We excluded studies and reports if they assessed the application of HBOT on other medical conditions such as diabetic ulcers, traumatic brain injury, etc. We also excluded studies which were of minimal importance to the topics, including reviews, editorials or letters without original data. We extracted the following data/information from each individual study: the first author’s last name, publication year, country, type of study, type of SSIs, study population, condition of HBOT procedure (pressure (ATA)/exposure time (min)), main findings, and conclusion (Table 1). Furthermore, we searched the above databases to identify studies describing the mechanisms of action of HBOT against SSIs, as well as complications and side effects of HBOT.

## 5. Results and Discussion

### 5.1. Application of HBOT in Surgical Site Infections

Initially, 359 relevant articles were retrieved. After screening of titles and abstracts and an in-depth review of full texts, we found 14 studies that used HBOT to treat different types of SSIs (Table 1). We categorized these studies based on the type of SSI or surgery: (1) sternal wound infection following cardiac surgery, (2) SSIs following neuromodulation or neuro-muscular surgery, and (3) SSIs following the male-to-female gender affirmation surgery (urogenital surgery).

#### 5.1.1. Application of HBOT in Sternal Wound Infection Infections

In the earliest report, Petzold et al. [54] used HBOT as an adjunct to local surgical treatment to treat an established sternal infection in an immuno-suppressed patient who developed presternal fat necrosis and subsequent sternal osteomyelitis two months after an orthotopic heart transplantation. Two areas of wound dehiscence developed. Conventional measures, including local debridement, sternal wire removal, and antiseptic irrigation, were applied in this case. After these measures, one wound was completely closed, but in the next weeks another wound showed only a slight tendency for further granulation and the purulent secretions increased. At that time, the physicians decided to apply HBOT for 40 sessions, each 90 min long and under 240 kPa of O_2_ pressure. With HBOT treatment, rapid healing was observed and the wound closed and was completely epithelialized. In a retrospective review of 27 cases of sternal infection treated over a 2-year period, Riddick et al. [65] reported that length of hospital stays and the readmission rate were reduced in patients that received HBOT; however, the authors did not perform statistical analysis to support these findings. In another retrospective review, De Feo et al. [66] compared the effects of conservative antibiotic therapy (group A) and aggressive surgical management (early debridement, removal of wires, and closed irrigation) in combination with granulated sugar and HBOT (group B) on morbidity and mortality following post-cardiotomy deep sternal wound infection. Although this study reported that morbidity and mortality related to deep sternal wound infection were significantly lower in group B, the authors made no conclusions about the specific benefits of HBOT. In line with study [66], Siondalski et al. [55] conducted a retrospective review of 55 patients over a 5-year period. The management plan consisted of aggressive surgery in combination with 20–40 HBO treatments. The authors conclude that the combination of aggressive surgical treatment and HBOT can improve the clinical outcome of patients with sternal infection.

Barili et al. [56], in a prospective trial, assessed the effect of HBOT on organ/space sternal SSIs following cardiac surgery that required sternotomy. Of the 32 participants in their study, 14 patients received HBOT, and 18 patients who did not consent to HBOT served as controls. The duration of infection was similar in the HBOT and control groups (31.8 ± 7.6 vs. 29.3 ± 5.7 days, respectively, *p* = 0.357). The relapse rate of SSI was significantly lower in the HBOT group (0% vs. 33.3%, *p* = 0.024). Furthermore, total hospital stays (52.6 ± 9.1 vs. 73.6 ± 24.5 days, *p* = 0.026) and the duration of intravenous antibiotic use (47.8 ± 7.4 vs. 67.6 ± 25.1 days, *p* = 0.036) were both significantly shorter in the HBOT group compared with the controls. The authors concluded that HBOT is a valuable addition to the techniques available for physicians to manage and treat postoperative organ/space sternal SSIs. Furthermore, Yu et al. [59] evaluated the effect of HBOT on sternal infection and osteomyelitis following median sternotomy. They included 12 patients: six received conventional therapy (debridement and antibiotic treatment), and six others received additional HBOT plus conventional therapy. Comparisons of the data between the two study groups revealed that the length of stay in the ICU, duration of invasive and noninvasive positive pressure ventilation, and hospital mortality were all significantly lower in patients with additional HBOT as compared to patients without HBOT. In another retrospective study of 18 patients undergoing HBOT after coronary artery bypass surgery, Egito et al. [60] demonstrated that HBOT used as an adjunctive therapy for the treatment of mediastinitis patients after CABS had favorable clinical results. Litwinowicz1 et al. [62] retrospectively assessed the effects and usefulness of additional HBOT in 10 patients with deep sternal wound infection (DSWI) after cardiac surgery. After four weeks of treatment, their findings revealed that HBOT used as an adjunct therapy was effective in treating 80% of patients with DSWI, with no complications observed. A retrospective study on children [64] showed that multimodality therapy, including incision and drainage and negative pressure wound therapy combined with HBOT and appropriate antibiotics, could be very helpful for the successful management of complex DSWI in the pediatric population after congenital heart surgery.

#### 5.1.2. Application of HBOT in SSIs following Neuromodulation or Neuro-Muscular Surgery

There are two studies indicating that HBOT could be a useful additional therapy with minimal side effects for the treatment or prevention of deep SSIs in complicated spine abnormalities in high-risk neuromuscular cases [58,61]. Larsson et al. evaluated possible benefits of HBOT in the treatment of deep postoperative SSIs in six high-risk pediatric patients with neuromuscular spine deformity. Their findings indicated that all infections were resolved, and a satisfactory correction with a balanced spine and radiologically healed fusion was achieved in all cases within three months. The implants were neither removed nor changed in any of the patients as a result of infection during the study period. Wound healing and normal blood tests were achieved within 4 months [58]. Inanmaz et al. demonstrated that HBOT prophylaxis in patients undergoing neuro-muscular scoliosis surgery can reduce the incidence of SSIs and improve the wound healing process [61]. Moreover, Bartek Jr. et al. applied HBOT as adjuvant treatment for hardware-related infections in neuromodulation. The findings of these studies indicated a potential benefit of adjuvant HBOT in the treatment of hardware-related infections in neuromodulation, diminishing the need for hardware removal and treatment interruption [63].

#### 5.1.3. Application of HBOT in SSIs following the Urogenital Surgery

According to our literature search, only one study by Stizzo et al. [21,67] assessed the effects of HBOT as an adjuvant treatment for SSIs after male-to-female gender affirmation surgery. In this study, 33 patients were enrolled: 15 received HBOT, while the remaining 18 patients belonged to the non-HBOT group. The results indicated that complete wound healing was not significantly different between the two groups, but the duration of antibiotic therapy, perineal drain time, bladder catheter time, and hospital stay were significantly lower in the HBOT group. The authors suggested using HBOT as an adjuvant treatment for SSIs in patients undergoing male-to-female gender affirmation surgery.

### 5.2. The Mechanisms of Action of Hyperbaric Oxygen Therapy (HBOT)

There is sufficient evidence suggesting HBOT as a useful approach in the treatment of different types of infections, either alone or as a supplement therapy, especially for deep and recalcitrant infections associated with hypoxia or induced by aerobic or anaerobic MDROs [68,69]. HBOT considerably increases the levels of O_2_ concentration in blood and damaged tissues, leading to several of the physiologic effects that help wound healing [39,70]. These physiologic effects include intravascular and tissue gas bubble reduction, vasoconstriction, improved oxygenation, modulation of inflammation and immune function, angiogenesis, and increased antimicrobial activity [39].

**Figure 2:**
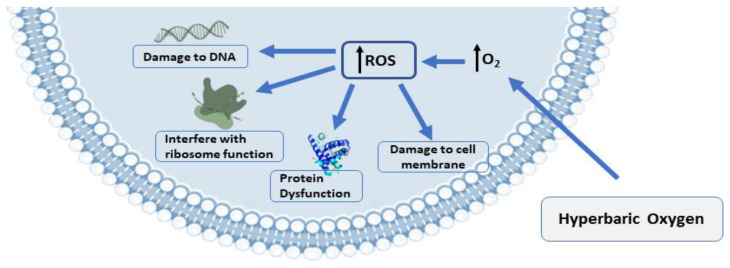
The presence of ROS production and biological targets in microbial growth: ROS production is the antibacterial mechanism of hyperbaric oxygen treatment (HBOT). DNA, proteins, and lipids are the targets of ROS’s damaging effects on cells. Abbreviations: ROS, reactive oxygen species.

**Figure 3:**
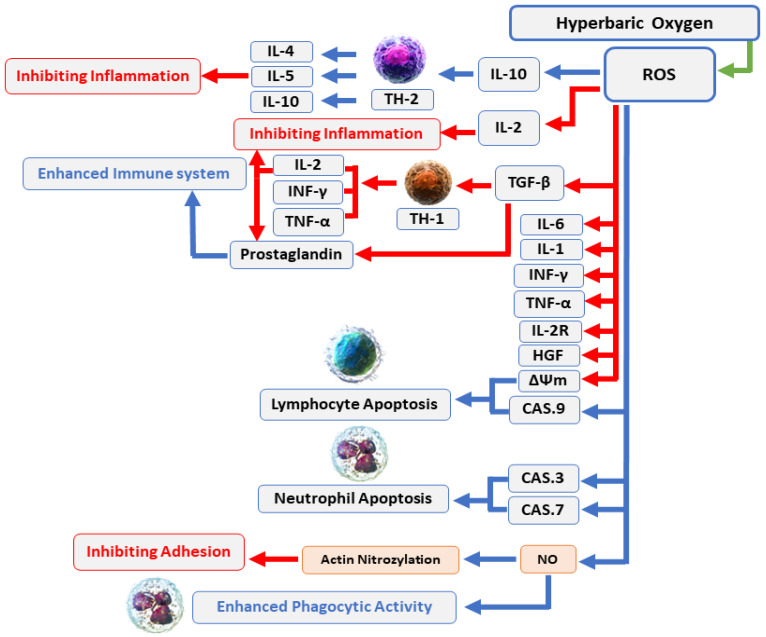
HBOT enhances the immune system’s antimicrobial effects: Increased O_2_ levels during HBOT have a variety of biological effects, including suppression of proinflammatory mediators, transitory reduction in the CD4:CD8 T cell ratio, and stimulation of lymphocyte and neutrophil death through caspase-3-, caspase-7-, and caspase-9-dependent mechanisms. In general, these effects can boost the antibacterial processes of the immune system and infection recovery. Abbreviations: ROS, reactive oxygen species; IL, interleukin; INF, interferon; TNF, tumor necrosis factor; CAS, caspase; NO, nitric oxide.

Comprehensive details about the mechanisms associated with antimicrobial activity and wound healing using HBOT are well-described in previous published reviews [71,72,73,74,75,76]. Briefly, the mechanisms related to the antimicrobial activity of HBOT could be divided into three main domains, including: (1) Direct antimicrobial or bactericidal effects by the formation of reactive oxygen species (ROS) [18]. HBOT can result in increased levels of ROS cells and eliminate the desired condition for bacterial agents that lack antioxidant defense pathways [75]. The antimicrobial activity of ROS is a dose-dependent mode of effect [71,77] and the main cellular targets of ROS are DNA, RNA, proteins, and lipids (Figure 2) [78,79]. (2) Immunomodulatory effects of HBOT that increase the antimicrobial effects of the immune system (Figure 3) [71]. HBOT has anti-inflammation effects by altering the expression of proinflammatory cytokines and other regulators of inflammation [80,81,82,83] and this anti-inflammatory effect has been reported to play an important role in reducing tissue damage and infection development [71]. HBOT also has a trigger effect on neutrophil migration to the site of infection via suppression of neutrophil beta-2 integrin (Mac-1 (CD11b/CD18)) activity [84,85,86]. As is well established, the O_2_ level of the environment is a critical factor for the antibacterial activity of neutrophils; therefore increased levels of O_2_ in the tissue environment after HBOT evidently increase the phagocytic and bactericidal activity of neutrophils [87]. (3) Additive or synergistic effects of HBOT with antibiotics. HBOT is generally applied as an adjuvant treatment in combination with antibiotic therapy in the treatment of infections; therefore, hyperoxia induced by HBOT may improve the activity of antibiotics [59,88,89]. It is suggested that the efficiency of some types of antibiotics, such as β-lactams, quinolones, and aminoglycosides, may influenced by the presence of O_2_ [71,90]. Experimental studies showed that HBOT used as a supplementary therapy improved the effects of tobramycin or cefazolin on Staphylococcus aureus [90,91]; vancomycin, teicoplanin, and linezolid on methicillin-resistant *S. aureus* [88]; and imipenem or ciprofloxacin on *Pseudomonas aeruginosa* [92,93].

### 5.3. The Complications and Side Effects of HBOT

Alongside the beneficial clinical effects of HBOT, several side effects and complications have also been described [94,95,96,97]. The two most frequent complications are middle ear barotrauma (MEB) and claustrophobia [95,97,98]. Patients suffering from MEB have ear pain, difficulty with ear equalization, a feeling of pressure, and, in rare cases, rupture of the tympanic membrane with a conductive hearing deficit [96,98]. Sinus/paranasal, pulmonary, and dental barotrauma are other common complications [96,97]. Other rare complications associated with HBOT are related to the toxic effects of oxygen and include myopia and cataracts, decompression sickness, hyperoxic myopia,, retrolental fibroplasia, O_2_-induced seizures, pulmonary oxygen toxicity, pulmonary edema, blood pressure effects and hypoglycemia in diabetic patients [39,71,96,97].

## 6. Limitations

In this work, we have undertaken an exhaustive review of the potential use of HBOT in the treatment of SSIs; although we acknowledge that our review has some limitations. The main limitation is the lack of literature, especially randomised controlled trials focused specifically on the evaluation of HBOT on SSIs. Most of the published studies were on infections related to diabetic foot, necrotizing soft tissue, or burns. Moreover, the majority of reviewed studies had a low sample size, leading to low statistical power in these studies.

## 7. Conclusions and Future Perspectives

HBOT, as primary or adjunctive therapy, showed many advantageous effects in the treatment of several medical conditions, especially for wound healing and infections. Despite the lack of valid randomised controlled trials, retrospective studies and case reports showed beneficial effects of HBOT in the treatment of SSIs or other similar infections. Considering increasing trends in the incidence of MDROs, HBOT can be effective in the prevention, management, or treatment of acute or chronic infections induced by such pathogens. We suggest conducting more research, especially randomized clinical trials and longitudinal investigations, to better standardize the treatment as well as to determine the full benefits and possible side effects of HBOT. We also suggest the development of specific indications to specify the potential contraindications to receiving this therapy.

Currently, there are only 14 approved indications for HBOT, and necrotizing soft tissue infections are the only indication for infectious diseases. We encourage further studies to extend the possible uses of HBOT to other types of infections, including SSIs. The next steps in this area should include: (i) increase of patients’ and physicians’ knowledge about the advantages of HBOT; (ii) exploration of the barriers limiting the use of HBOT in the treatment of SSIs and other infections; and (iii) development of specific guidance for HBOT in the treatment of SSIs.

## Figures and Tables

**Table 1:** Overview of studies investigating the application of HBOT in treatment of surgical site infections.

Study	Type of Study	Type of Surgery	Type of SSI	Study Population Total; HBOT/Non-HBOT	Pressure (ATA)/Exposure Time (min)	Main Findings and Conclusion
Petzold et al. (1999) [54]	Case report	Cardiac surgery	Sternal SSI	1	2.40/90	* HBOT resulted in rapid healing and epithelialization of the wound * This was the first reported case of HBOT used for the treatment of deep sternal SSI in a heart transplant recipient.
Siondalski et al. (2003) [55]	Retrospective study	Sternotomy	Sternal SSI	55	2.50/90	* The Sternal SSI was cured in all patients treated with HBOT within an average of 8 weeks * There was no in-hospital death. * The combination of surgical treatment and HBOT could improve clinical outcome in patients with sterno-mediastinis and poststernotomy wound infection after cardiac surgery
Barili et al. (2007) [56]	Prospective trial	Cardiac surgery	Sternal SSI	32; 14/18	2–3/90	* Staphylococcus was the most common pathogen for both groups. * The duration of infection was similar in groups 1 and 2 (31.8 ± 7.6 vs. 29.3 ± 5.7 days, respectively, *p* = 0.357). * The infection relapse rate was significantly lower in group 1 (0% vs. 33.3%, *p* = 0.024). * The duration of intravenous antibiotic use (47.8 ± 7.4 vs. 67.6 ± 25.1 days, *p* = 0.036) and total hospital stay (52.6 ± 9.1 vs. 73.6 ± 24.5 days, *p* = 0.026) were both significantly shorter in group 1. * HBOT could be a valuable addition to the armamentarium available to physicians treating postoperative organ/space sternal SSI.
Sun et al. (2008) [57]	Case report	Cardiac surgery	Sternal SSI	1	2.50/90	* After nine weeks, the sternal wound was healed and completely epithelialized. * HBOT with Topical Negative Pressure (TNP) dressing is a good alternative method for patients who cannot tolerate or refuse to receive any surgical reconstruction.
Larsson et al. (2011) [58]	Retrospective study	neuro-muscular scoliosis surgery	Deep wound infection	6	2.50/3 × 25	* All infections were resolved and wound healing occurred within an average of 3 months. * Side effects of HBO treatment were minor. * HBOT is a safe and potentially useful adjuvance treatment for early deep postoperative infections in complex situations with spinal implants in high-risk paediatric patients.
Yu et al. (2011) [59]	Retrospective study	Cardiac surgery	Sternal SSI	12; 6/6	2.50/90	* HBOT did not cause any treatment-related complication in patients receiving this additional treatment. * Comparisons of the data between two study groups revealed that the length of stay in ICU (8.7 ± 2.7 days vs. 48.8 ± 10.5 days, *p* < 0.05), duration of invasive (4 ± 1.5 days vs. 34.8 ± 8.3 days, *p* < 0.05) and noninvasive (4 ± 1.9 days vs. 22.3 ± 6.2 days, *p* < 0.05) positive pressure ventilation, and hospital mortality (0 case vs. 3 cases, *p* < 0.05) were all significantly lower in patients who received additional HBOT, as compared to patients who did not receive HBOT. * In addition to primary treatment with debridement and antibiotic use, HBOT may be used as an adjunctive and safe treatment to improve clinical outcomes in patients with sternal infection and osteomyelitis after sternotomy and cardiothoracic surgery.
do Egito et al. (2013) [60]	Retrospective study	Coronary artery bypass surgery	Mediastinitis	18	2.50/90	* There was only one hospital death, 7 months after the oxygen therapy, which was caused by sepsis and was unrelated to HBOT. * HBOT was well-tolerated. * HBOT used as an adjunctive therapy for treatment of mediastinis patients after CABS resulted in favorable clinical outcomes.
Inanmaz et al. (2014) [61]	Retrospective study	neuro-muscular scoliosis surgery	Deep wound infection	42; 18/24	2.40/90	* The overall incidence of infection in the whole study group was 11.9% (5/42). * The infection rate in the P-HBO and the control group were 5.5% (1/18), and 16.6% (4/24) respectively. * The use of HBO was found to significantly decrease the incidence of postoperative infections in neuromuscular scoliosis patients. * This study indicated that HBOT is a safe and potentially useful supplement which can be used to prevent postoperative deep infections in complex spine deformity in high-risk neuromuscular patients.
Litwinowicz et al. (2016) [62]	Retrospective study	Cardiac surgery	Sternal SSI	10	2.50/92	After 4 weeks of HBOT, seven (70%) patients presented complete wound healing with fibrous scar formation. * One patient qualified for another cycle of HBOT with twenty additional sessions, and after that complete wound healing with fibrous scar formation was observed. * In 2 cases, patients received 5 and 19 of 20 sessions; however, the HBOT course was interrupted because of the patients did not qualify for HBOT. * HBOT, as an additional therapy in DSWI, was successful in 80% of cases, and no complications were observed.
Bartek Jr et al. (2018) [63]	Retrospective study	Neuromodulation	Hardware-related infection	14	2.0–2.8/75	* Twelve out of fourteen events of hardware-related infection were successfully treated without hardware removal (86%). * Two patients treated twice with HBOT on two time-independent occasions could retain their hardware in both cases. * Hardware was removed following HBOT failure in two infection events, with long-term infection control achieved in all patients. * Furthermore, an intrathecal pump malfunction caused by HBOT at 2.8 bars was observed, leading to a change in the manufacturer’s guidelines. * This study indicates a potential benefit of adjuvant HBOT in the treatment of hardware-related infections in neuromodulation, diminishing the need for hardware removal and treatment interruption.
Copeland et al. (2018) [64]	Retrospective study	Cardiac surgery	Sternal SSI	53		* The time to discharge for patients readmitted with infected sternotomies was 7.71 (+7.339) days (range: 2–39 days). * The mean duration of time for the wounds to heal with the use of Negative Pressure Wound Care Therapy (NPWT) alone was 31.50 (+12.12) days (range: 21–42 days, median: 31.5 days). * The healing time for wounds treated with HBO was a mean of 35 (+9.90) days (range: 28–42 days; median: 35 days). * The duration of HBOT was an average of 16.17 (+8.99) days (range: 5–35 days), and the average number of HBO treatments was 22.6 (+11.06). * The time to heal for patients who had both NPWT and HBO therapy was 42.88 (+24.94) days (range: 21–98 days, median: 42 days). * The results of this study demonstrate that the multimodality therapy of incision and drainage, and NPWT combined with HBOT and appropriate antibiotics, is successful for management of complex deep sternal wound infections in the pediatric population after congenital heart surgery.
Stizzo et al. (2022) [21]	Retrospective study	male-to-female gender affirmation surgery (MtF-GAS)	SSI	33; 15/18	2.2–3.0/90	* Complete wound healing was obtained in all 15 patients (100%) of the HBOT group and 17 patients (94.4%) in the non-hyperbaric oxygen therapy group (*p* = 0.35). * Duration of antibiotic therapy, perineal drain time, bladder catheter time, and hospital stay were significantly lower in the HBOT group (*p* < 0.05). * This study indicated a role for HBOT as an adjuvant treatment for SSIs in patients undergoing MtF GAS.

## Data Availability

No new data were created or analyzed in this study. Data sharing is not applicable to this article.
